# Development and validation of prediction models for diabetic retinopathy in type 2 diabetes patients

**DOI:** 10.1371/journal.pone.0325814

**Published:** 2025-07-10

**Authors:** Shadi Naderyan Feˈli, Mohammad Hassan Emamian, Mehdi Yaseri, Hamid Riazi-Esfahani, Hassan Hashemi, Akbar Fotouhi, Kamran Yazdani

**Affiliations:** 1 Department of Epidemiology and Biostatistics, School of Public Health, Tehran University of Medical Sciences, Tehran, Iran; 2 Ophthalmic Epidemiology Research Center, Shahroud University of Medical Sciences, Shahroud, Iran; 3 Farabi Eye Hospital, Tehran University of Medical Sciences, Tehran, Iran; 4 Noor Ophthalmology Research Center, Noor Eye Hospital, Tehran, Iran; Kerman University of Medical Sciences Physiology Research Center, IRAN, ISLAMIC REPUBLIC OF

## Abstract

**Background and objective:**

Prediction models enable healthcare providers to perform early risk stratification. This study aimed to develop and internally validate prediction models for 5- and 10-year risks of developing diabetic retinopathy (DR) in the Iranian individuals with type 2 diabetes.

**Methods:**

This study utilized data from individuals with diabetes involved in the Shahroud Eye Cohort Study (ShECS), a prospective cohort study in Iran. The initial phase of ShECS began in 2009, with the second and third follow-up phases occurring in 2014 and 2019, respectively. Logistic regression developed prediction models, with bootstrap validation assessing internal validity. Model performance was evaluated using the discrimination and calibration.

**Results:**

A total of 637 individuals with diabetes (35.0% men, mean (SD) of age: 53.0 (6.3 years)) were diagnosed. The five-year cumulative incidence of DR was 25.3% (95%CI: 21.8, 29.0%), and 17.0% (95%CI: 13.3, 21.0%) based on the second and third phases, respectively, while 10-year cumulative incidence was 40.0% (95%CI: 35.8, 44.0%). Incorporating various predictors, six models were developed with three recommended prediction models. Using mean blood pressure (MBP), non-fasting blood glucose (BG), and diabetes duration, Model-1 predicts 5-year risk indicating good calibration and discrimination with a c-statistic of 0.773 after bootstrap validation. The optimal statistical threshold was a predicted probability of 0.24. Model-2 predicts a 10-year risk incorporating diabetes duration, MBP, and BG, with a good calibration and a c-statistic of 0.687 after bootstrap validation showing moderate discrimination. The optimal statistical threshold was a predicted probability of 0.32. Model-3 predicts the 5-year risk using diabetes duration, MBP, glycated hemoglobin, high-density lipoprotein, triglycerides, and fasting blood glucose, showing good calibration and a c-statistic of 0.735 after bootstrap validation, indicating good discrimination. The optimal statistical threshold was a predicted probability of 0.20.

**Conclusion:**

Three prediction models with satisfactory performance were obtained using readily available predictors.

## Introduction

Diabetic retinopathy ‌(DR) is one of the most common complications of diabetes. Based on a meta-analysis of 59 population-based studies on the individuals with diabetes, the global prevalence of diabetic retinopathy, vision-threatening DR, and clinically significant macular edema was reported to be 22.3%, 6.2%, and 4.1%, respectively [1]. An analysis of a comprehensive nationwide U.S. registry showed that out of 53,535 eyes from newly diagnosed patients with DR who had good vision, 678 eyes (1.3%) progressed to sustained blindness over an average follow-up time of approximately 1.8 years [2].

Prediction models can assist clinicians in making individualized decisions regarding patient management by combining a set of patient characteristics as predictor variables [3,4]. Applications of DR prediction models are comparable to those of other predictive models. One of these models is the prediction model by Gong et al., which was developed and validated using data from a Chinese cohort study [5], and Nugawela et al. models, which were developed using a longitudinal study from the UK and externally validated using cohorts from Wales, the UK and India [6]. The factors such as age, sex, body mass index (BMI), systolic blood pressure (SBP), diabetes duration, glycated hemoglobin (HbA1c), antidiabetic medication use, and history of retinopathy were introduced as predictors of DR or sight threatening DR. To the best of our knowledge, only model available in Iran was developed by Azizi-Soleiman et al. [7]. However, the model was based on data from a cross-sectional study, which was inappropriate for prediction. Furthermore, while it is sometimes feasible to use an already-developed predictive model, it is preferred to create a tailored one for each particular population owing to variations in racial and ethnic backgrounds, the distribution of risk factors, and the incidence rate of outcomes [8,9]. Furthermore, a prediction model established using readily available predictors can be efficiently used in primary healthcare settings.

Considering the initial stages of DR are asymptomatic [10], early identification of DR risk (probability) can justify timely diagnosis and referral for a comprehensive ophthalmological examination. This study aimed to develop and internally validate models to predict 5- and 10-year risks of developing DR among Iranian individuals with type 2 diabetes mellitus (T2DM), which can enable primary healthcare providers, and physicians to perform early risk stratification of these patients.

## Materials and methods

### Study design and participants

This study was conducted using the data collected over 10 years in three phases of Shahroud Eye Cohort Study (ShECS). ShECS is a population-based prospective cohort study for detecting risk factors of visual impairments and major eye conditions started in 2009 in Shahroud, Iran. The first phase of the ShECS was completed by 5,190 participants, ages 40–64, who were selected using a random cluster sampling technique. All participants of the first phase were invited to return for a follow-up examination after a period of 5 and 10 years. Therefore, phases two and three of the follow-up were carried out in 2014 and 2019, respectively. The details of the study design were reported elsewhere [11]. Data for current study were accessed on 2022/06/25. The data collection contained no personal identifiers for the study participants.

Individuals with diabetes diagnosed in the first phase of ShECS were included in the study. Those with DR in the baseline phases were not included in the development of prediction models. Therefore, participants with DR in the first and second phases of the ShECS were excluded when the models were being developed.

TRIPOD (Transparent Reporting of a multivariable prediction model for Individual Prognosis Or Diagnosis) Statement was followed for reporting this study [12]. This study, which was part of a Ph.D. dissertation in epidemiology, was approved by Institutional Review Board of Tehran University of Medical Sciences (IRB no. IR.TUMS.SPH.REC.1401.048). All three phases of ShECS were separately reviewed and approved by the Institutional Review Board of Shahroud University of Medical Sciences. Moreover, a written informed consent was obtained from each of the participants.

### Measurements

At the first phase of the ShECS, a non-fasting blood glucose level of 200 mg/dL or higher and/or taking glucose-lowering drugs were considered diabetes [13]. Considering low prevalence of type 1 diabetes in adults, we considered all individuals with diabetes as type 2 [14].

#### Outcome.

To determine if DR was present in the first phase of the ShECS, participants were asked about their DR diagnosis status. Morovere, participants recieved two occular examinations in the first and second phase of the ShECS. During these stages of the ShECS, ophthalmologists used direct or indirect ophthalmoscopy to examine the retina. To diagnose DR, fundus imaging was also used. A Nidek AFC-230 Fundus Camera (Nidek, Chiyoda-ku, Japan) was used for fundus photography.

#### Predictors.

As a review of the literature is a common approach for narrowing down the list of candidate predictors [15], the most relevant candidate predictors, that could be readily available in clinical practice and did not require an ophthalmological examination, were identified based on a review of the literature. All measurements were based on the ShECS protocol.

Participants were asked about their birth date, smoking status (cigarette, pip, or hookah), and diabetes duration (time since diabetes diagnosis) via a face-to-face interview [11]. The participants’ sex was determined based on the investigator’s observations. Trained nurses measured SBP and diastolic blood pressure (DBP) using a digital blood pressure sphygmomanometer [16]. Mean blood pressure (MBP) was defined as (2 × DBP + 1 × SBP)/3. Fasting blood glucose (FBG), HbA1c, and blood lipid levels were measured after 12–14 hours of fasting [17]. Non-fasting blood glucose (BG) was assessed [18]. Body weight was measured using digital scales, and body height was measured in a standing position [19] and BMI was subsequently calculated as the ratio of weight (in kilograms) to the square of height (in meters). Waist circumference (WC) was measured at the highest part of the iliac crest using a non-stretchable tape [17]. A non-elastic tape was used to measure hip circumference (HC) in the biggest pelvic circumference region while the subject was standing (personal communication). Then, WC divided by HC was used to compute the waist-to-hip ratio (WHR). Pulse rate was recorded as number of beats per minute (bpm) using a digital sphygmomanometer (personal communication).

### Statistical analyses

#### Descriptive statistics.

For categorical variables, frequencies and percentages were used as the descriptive measures. Quantitative variables were described using mean, and standard deviation. In cases where quantitative variables were not near a normal distribution, the median and interquartile range were reported along with the mean and standard deviation.

#### Development and validation of the prediction models.

**Fig 1:**
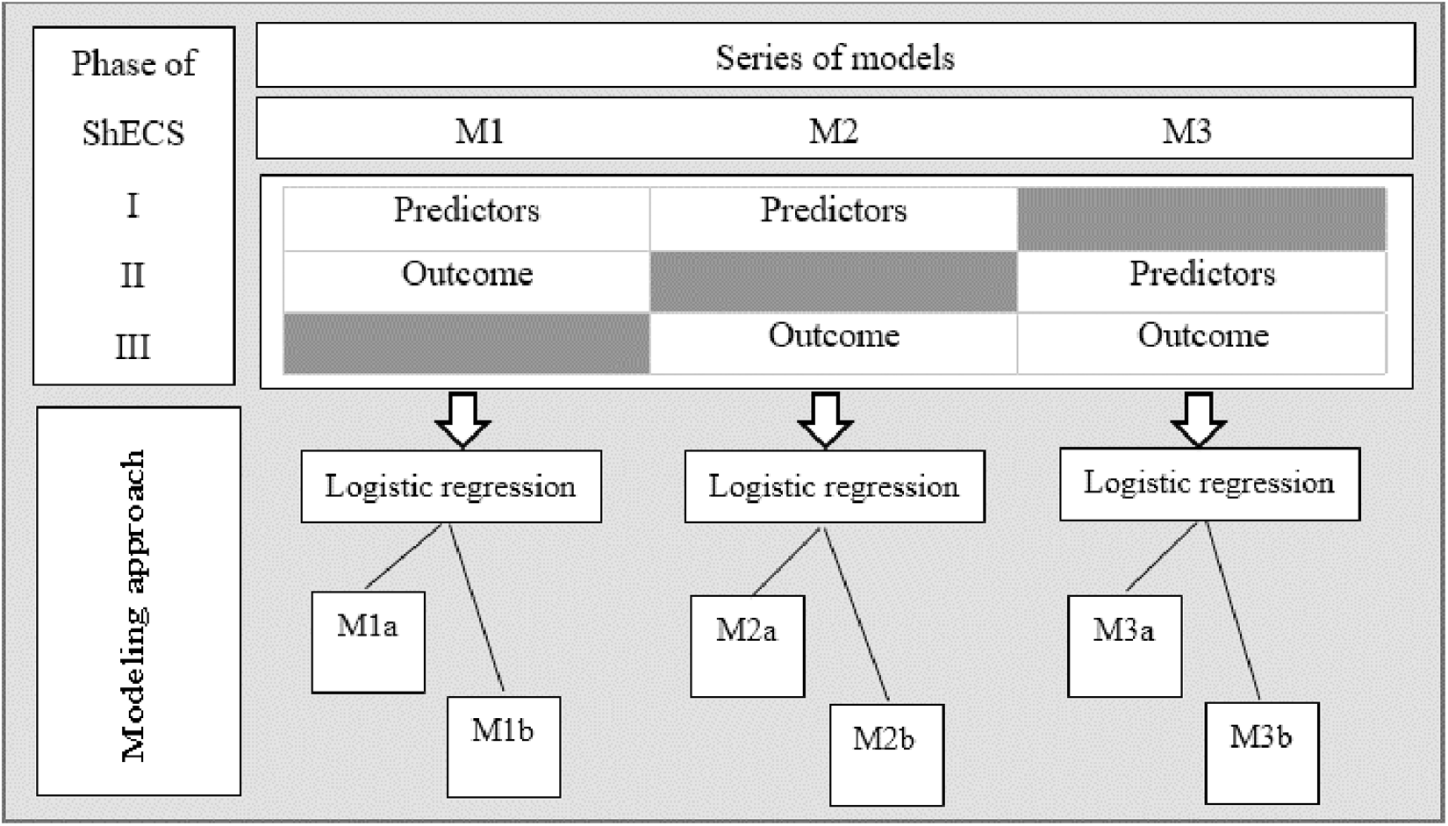
The model development process. Predictors considered for developing M1 included: sex, smoking, age, diabetes duration, blood pressure, body mass index, and non-fasting blood glucose. Predictors considered for developing M2 included: sex, smoking, age, diabetes duration, blood pressure, body mass index, and non-fasting blood glucose. Predictors considered for developing M3 included: sex, smoking, age, diabetes duration, blood pressure, body mass index, HbA1c, total cholesterol, high-density lipoprotein cholesterol, triglycerides, fasting blood glucose, pulse rate, abdomen circumference, hip circumference, abdominal to hip ratio. a: systolic and diastolic blood pressure were included as separate predictors. b: mean blood pressure ((2*DBP + 1*SBP)/3) was included.

Because not all the predictors were measured simultaneously during the phase one of the ShECS, three separate models were developed, as illustrated in the Fig 1. Models M1 and M3 predict 5-year risk of DR, and M2 predicts the 10-year risk. Log-binomial, logistic, and Poisson regression models with robust standard error estimates were used to achieve the optimal risk prediction model. However, only the results of the logistic regression were presented since the log-binomial regression was unable to converge and there was no simple way to evaluate the calibration of the Poisson models. The predictors included in each model are described in the Fig 1. Since SBP in M1, DBP in M2, and both blood pressures in M3 were statistically significant predictors, each of the three models was fitted separately regarding SBP-DBP and MBP to utilize all available information on the blood pressure variable. We used a complete case analysis so that participants with missing values were excluded from the model-building and model-validation processes.

**Model development.**

A univariable regression analysis was conducted, following the criterion of a P-value<0.2, to examine unadjusted associations. However, key predictors were not excluded solely based on statistical non-significance in the univariable analysis [20]. Subsequently, a multivariable analysis was performed using a backward elimination approach, focusing on the remaining predictors. In multivariable regression, predictors and interaction terms with P-value<0.05 were retained in the final models.

We considered a generalized additive model (GAM) and corresponding plots in univariable and multivariable analyses to check the nonlinearity of associations between outcome and continuous predictors. An appropriate polynomial term was supposed to be included in the model if there was evidence of a nonlinear association; if not, the predictors were thought to be linear. To find potentially influential observations, a modified Cook’s distance plot was used. If any influential observations were found, a sensitivity analysis was carried out, in which the final model was repeated without the influencing points. To detect multicollinearity, which is a high correlation among predictors, we used the variance inflation factor (VIF). A VIF above 10 was considered as high collinearity.

**Model performance measures.**

- Discrimination, that is the ability of a prediction model to discriminate among the patients with and without future outcomes was quantified using Harrell’s concordance (c) statistic, which was equal to the area under the Receiver Operating Characteristic curve (AUC).
  - Calibration, which specifies the agreement between the observed, and predicted outcomes of all participants, was graphically illustrated using calibration plots and statistically tested using Hosmer–Lemeshow goodness-of-fit test. A p-value of greater than 0.05 indicated a good fit. Moreover, the expected (predicted) to observed (E/O) ratio was calculated by dividing the expected to observed outcome risks.

**Model validation.**

- Three distinct approaches were applied to assess internal validation of final models: 1) for apparent validation, performance measures were assessed directly on the data used to drive the models, resulting in optimistic performance estimates, 2) following the approach outlined by Kuhn and Johnson [21] cross-validated performance measures were derived using a stratified 10-fold cross-validation method, 3) following the approach outlined by Steyerberg, performance measures were additionally obtained using a bootstrap validation with 200 bootstrap samples, that reflect future performance in new patients [22]. However, there was a significant amount of random error in the performance measures in the 10-fold cross-validation since there weren’t many outcomes in certain folds. Rather, more stable measures were established using bootstrap validation. Therefore, only performance measures using bootstrap validation approach were reported.
- We compared the apparent c-statistic and calibration plots with those after bootstrap validation. In the case of a considerable drop in performance measures, it was intended to recheck the models in terms of the number of included predictors in relation to the sample size.

#### Decision-curve analysis.

A decision curve was formed using net benefit (NB) on y-axis, and thresholds of predicted probabilities on x-axis. The NB was defined as (TP –w×FP)/N, where “w” indicated a weight equal to the odds of selected threshold (Pt/1-Pt), which is considered as the harm to benefit ratio of treatment. For example, at the threshold of 10%, the false-positives is valued at one-ninth of true-positives. “Pt” is the threshold probability, “TP” was the number of true-positives, “FP” is the number of false-positives, and N was the total number of subjects [23]. To determine the model line, we plotted NBs against a broad range (0–1) of predicted probability thresholds. Additionally, two additional lines were drawn to illustrate the default strategies of “treat none” and “treat all” without the use of the prediction model. We regarded NB as zero for the “treat none” line, and we drew the “treat all” line depending on the thresholds and event rate [24,25].

Using the decision curve of each model, the interval between the intersection points of model’s line with both lines of default strategies was considered the reasonable interval of thresholds for prediction. As is usual, the statistical optimum decision threshold was determined based on Youden’s J statistic (J = model sensitivity +model specificity−1). Decision curves were plotted using both apparent and bootstrap validation approaches.

Regarding the use of cluster sampling, robust standard errors and p-values were considered to correct the design effect. STATA_17_ (StataCorp, USA), and R_4.2.3_ statistical software were used to perform the statistical analyses.

## Results

### Characteristics of the participants

A total of 5,190 individuals, of whom 5,183 participants underwent blood glucose tests completed the first phase of the ShECS. Among them, 637 were diagnosed with diabetes, representing a 12.3% prevalence. The mean age of those with diabetes was 53.0 years (standard deviation: 6.3 years), and 35.0% and 65% of them were men and women, respectively. As a baseline, 24 (3.8%) and 148 (23.2%) participants with DR in the first and second phases of the ShECS, respectively, were not included in the analyses. There were 148 and 64 incident cases of DR during the second and third phases, respectively. The five-year cumulative incidence of DR based on the second and third phase was 25.3% (95%CI: 21.8, 29.0%), and 17.0% (95%CI: 13.3, 21.0%), respectively. Moreover, the 10-year cumulative incidence was 40.0% (95%CI: 35.8, 44.0%). The predictors measured in each phase of ShECS are described in Table 1.

**Table 1:** Characteristics of the participants in each phase of the Shahroud Eye Cohort Study.

Participant’s characteristics	Phase 1 (n = 637)	Phase 2 (n = 557)
Male sex	223 (35.0)	219 (37.7)
Smoking	45 (7.1)	51 (9.2)
Age (years)	53.0 ± 6.3	57.9 ± 6.1
Diabetes duration (years)	5.4 ± 5.6 4.0 [9.0]	10.1 ± 5.5 8.0 [7.0]
SBP (mmHg)	136.9 ± 22.2	136.5 ± 21.8
DBP (mmHg)	80.6 ± 12.0	80.7 ± 12.1
MBP (mmHg)	99.4 ± 13.8	95.1 ± 12.4
BMI (kg/m^2^)	29.6 ± 4.7	29.8 ± 4.9
BG (mg/dL)	240.1 ± 111.7 227.0 [140.0]	*NA*
HbA1c (%)	*NA*	7.6 ± 1.7
TC (mg/dL)	*NA*	184.4 ± 45.3 180.5 [55.0]
HDL-C (mg/dL)	*NA*	40.6 ± 11.5
TG (mg/dL)	*NA*	207.5 ± 119.2 178.0 [113.5]
FBG (mg/dL)	*NA*	168.2 ± 68.7 154.5 [84.0]
PR (bpm)	*NA*	80.6 ± 12.0
WC (cm)	*NA*	104.3 ± 11.5
HC (cm)	*NA*	106.0 ± 11.1
WHR (cm)	*NA*	0.1 ± 0.3, 0.1 [0.1]

Data are expressed as numbers (percentages), mean ± standard deviation, median [interquartile range]. Predictors of Phase 3 were not reported, as they were not used in the present study. Abbreviations: SBP: Systolic blood pressure, DBP: Diastolic blood pressure, MBP: mean blood pressure, BMI: Body mass index, BG: Non-fasting blood glucose, HbA1c: Glycated hemoglobin, TC: Total cholesterol, HDL-C: high-density lipoprotein cholesterol, TG: Triglycerides, FBG: Fasting blood glucose, PR: Pulse rate, WC: waist circumference, HC: Hip circumference, WHR: Waist-to-hip ratio, NA: Not applicable

### Diabetic retinopathy prediction models

Based on GAM plots no nonlinear association between predictors and outcomes was observed in any of the univariable or multivariable models. Besides, after performing sensitivity analyses, no outlier observations were identified as influential. Moreover, no severe multicollinearity was observed among the predictors (the results of these assumptions have not been reported).

The unadjusted univariable associations between candidate predictors and DR are reported in Table 2. Final risk prediction models are reported in Table 3 and 4.

**Table 2:** Unadjusted association between each candidate predictor and outcome using univariable logistic regression.

Predictors	M1	M2	M3
Sex^a^	−0.099 (0.617)	−0.243 (0.212)	0.115 (0.679)
Smoking^b^	0.253 (0.488)	−0.348 (0.428)	−0.138 (0.773)
Age (years)	**0.021 (0.171)**	−0.004 (0.767)	−0.007 (0.723)
Diabetes duration (years)	**0.120 (<0.001)**	**0.075 (<0.001)**	**0.044 (0.050)**
SBP (mmHg)	**0.013 (<0.001)**	**0.010 (0.020)**	**0.012 (0.080)**
DBP (mmHg)	−0.003 (0.712)	**0.013 (0.132)**	**0.018 (0.123)**
MBP (mmHg)	**0.010 (0.136)**	**0.015 (0.041)**	**0.019 (0.057)**
BMI (kg/m^2^)	**−0.031 (0.176)**	0.014 (0.543)	0.012 (0.704)
BG (mg/dL)	**0.005 (<0.001)**	**0.004 (<0.001)**	*NA*
HbA1c (%)	*NA*	*NA*	**0.546 (<0.001)**
TC (mg/dL)	*NA*	*NA*	−0.000 (0.874)
HDL-C (mg/dL)	*NA*	*NA*	**−0.029 (0.009)**
TG (mg/dL)	*NA*	*NA*	**0.001 (0.163)**
FBG (mg/dL)	*NA*	*NA*	**0.009 (<0.001)**
PR (bpm)	*NA*	*NA*	**0.025 (0.030)**
WC (cm)	*NA*	*NA*	0.007 (0.567)
HC (cm)	*NA*	*NA*	−0.000 (0.988)
WHR (cm)	*NA*	*NA*	1.644 (0.374)

Data are expressed as regression coefficient (P-value).

^a^ Males were the reference category.

^b^ Non-smokers were the reference category.

Abbreviations: SBP: Systolic blood pressure, DBP: Diastolic blood pressure, MBP: mean blood pressure, BMI: Body mass index, BG: Non-fasting blood glucose, HbA1c: Glycated hemoglobin, TC: Total cholesterol, HDL-C: high-density lipoprotein cholesterol, TG: Triglycerides, FBG: Fasting blood glucose, PR: Pulse rate, WC: Waist circumference, HC: Hip circumference, WHR: Waist-to-hip ratio, NA: Not applicable

**Table 3:** Prediction models with systolic and diastolic blood pressure as two distinct predictors, using multivariable logistic regression.

Predictors	M1 (n = 520) (missingness = 15.2%, outliers:1.1%)	M2 (n = 435) (missingness = 29.0%, outliers: 1.8%)	M3 (n = 326) (missingness = 33.0%, outliers:16.9%)
Intercept	−5.5445 (−7.0171, −4.0720), P < 0.001	−4.0152 (−5.6111, −2.4194), P < 0.001	1.7980 (−7.0696, 10.0665), P = 0.69
Sex^a^	*NA*	*NA*	*NA*
Smoking^b^	*NA*	*NA*	*NA*
Age (years)	*NA*	*NA*	*NA*
Diabetes duration (years)	0.1384 (0.0983, 0.1784), P < 0.001	0.0897 (0.0521, 0.1274), P < 0.001	0.1708 (0.0581, 0.2834), P = 0.003
SBP (mmHg)	0.0146 (0.006, 0.023), P < 0.001	*NA*	0.0533 (0.0129, 0.0937), P = 0.01
DBP (mmHg)	*NA*	0.0203 (0.0023, 0.0384), P = 0.03	−0.0659 (−0.1229, −0.0088), P = 0.02
BMI (kg/m^2^)	*NA*	*NA*	*NA*
BG (mg/dL)	0.0071 (0.0048, 0.0093), P < 0.001	0.0048 (0.0028, 0.0068), P < 0.001	*NA*
HbA1c (%)	*NA*	*NA*	−0.7913 (−1.6208, 0.0383), P = 0.06
TC (mg/dL)	*NA*	*NA*	*NA*
HDL-C (mg/dL)	*NA*	*NA*	−0.2856 (−0.4633, −0.1078), P = 0.002
TG (mg/dL)	*NA*	*NA*	−0.0213 (−0.0395, −0.0031), P = 0.02
FBG (mg/dL)	*NA*	*NA*	0.0423 (0.0150, 0.0697), P = 0.002
PR (bpm)	*NA*	*NA*	*NA*
WC (cm)	*NA*	*NA*	*NA*
HC (cm)	*NA*	*NA*	*NA*
WHR (cm)	*NA*	*NA*	*NA*
Diabetes duration*FBG	*NA*	*NA*	−0.0007 (−0.0015, −0.00004), P = 0.04
SBP*FBG	*NA*	*NA*	−0.0002 (−0.0004, −0.00005), P = 0.01
HbA1c* HDL-C	*NA*	*NA*	0.0329 (0.0106, 0.0552), P = 0.004
DBP*TG	*NA*	*NA*	0.0002 (0.00003, 0.0005), P = 0.03
HbA1c*FBG	*NA*	*NA*	*NA*
DBP*FBG	*NA*	*NA*	*NA*

Data are expressed as regression coefficient (95% confidence interval).

^a^ Males were the reference category.

^b^ Non-smokers were the reference category.

P: p-value, P < 0.05 was considered statistically significant, stars indicate the interaction between two predictors, Abbreviations: SBP: Systolic blood pressure, DBP: Diastolic blood pressure, BMI: Body mass index, BG: Non-fasting blood glucose, HbA1c: Glycated hemoglobin, TC: Total cholesterol, HDL-C: high-density lipoprotein cholesterol, TG: Triglycerides, FBG: Fasting blood glucose, PR: Pulse rate, WC: Waist circumference, HC: Hip circumference, WHR: Waist-to-hip ratio, NA: Not applicable

**Table 4:** Prediction models with mean blood pressure as a combination of systolic and diastolic blood pressure, using multivariable logistic regression.

Predictors	M1 (n = 520) (missingness = 15.2%, outliers: 5.6%)	M2 (n = 435) (missingness = 29.0%, outliers: 3.0%)	M3 (n = 326) (missingness = 33.0%, outliers: 3.0%)
Intercept	−5.2855 (−7.1112, −3.4598), P < 0.001	−4.2554 (−5.9538, −2.5571), P < 0.001	8.4990 (0.4733, 16.5251), P = 0.04
Sex^a^	*NA*	*NA*	*NA*
Smoking^b^	*NA*	*NA*	*NA*
Age (years)	*NA*	*NA*	*NA*
Diabetes duration (years)	0.1412 (0.1007, 0.1818), P < 0.001	0.0876 (0.0507, 0.1245), P < 0.001	0.1827 (0.06904, 0.2964), P = 0.002
MBP (mmHg)	0.0175 (0.0017, 0.0332), P = 0.03	0.0192 (0.0033, 0.0351), P = 0.02	−0.0389 (−0.0831, 0.0052), P = 0.08
BMI (kg/m^2^)	*NA*	*NA*	*NA*
BG (mg/dL)	0.0070 (0.0048, 0.0093), P < 0.001	0.0048 (0.0028, 0.0068), P < 0.001	*NA*
HbA1c (%)	*NA*	*NA*	−0.8821 (−1.6421, −0.1221), P = 0.02
TC (mg/dL)	*NA*	*NA*	*NA*
HDL-C (mg/dL)	*NA*	*NA*	−0.3027 (−0.4696, −0.1358), P = 0.004
TG (mg/dL)	*NA*	*NA*	−0.0214 (−0.0394, −0.0035), P = 0.02
FBG (mg/dL)	*NA*	*NA*	0.0117 (0.0018, 0.0216), P = 0.02
PR (bpm)	*NA*	*NA*	*NA*
WC (cm)	*NA*	*NA*	*NA*
HC (cm)	*NA*	*NA*	*NA*
WHR (cm)	*NA*	*NA*	*NA*
Diabetes duration*FBS	*NA*	*NA*	−0.0008 (−0.0016, −0.0001), P = 0.02
HbA1c* HDL-C	*NA*	*NA*	0.0345 (0.0137, 0.0553), P = 0.001
TG*MBP	*NA*	*NA*	0.0002 (0.00003, 0.0004), P = 0.02
TG*FBG	*NA*	*NA*	*NA*
TG*HDL-C	*NA*	*NA*	*NA*

Data are expressed as regression coefficient (95% confidence interval).

^a^ Males were the reference category.

^b^ Non-smokers were the reference category.

P: p-value, P < 0.05 was considered statistically significant, stars indicate the interaction between two predictors, Abbreviations: MBP: mean blood pressure, BMI: Body mass index, BG: Non-fasting blood glucose, HbA1c: Glycated hemoglobin, TC: Total cholesterol, HDL-C: high-density lipoprotein cholesterol, TG: Triglycerides, FBG: Fasting blood glucose, PR: Pulse rate, WC: Waist circumference, HC: Hip circumference, WHR: Waist-to-hip ratio, NA: Not applicable

### Model performance

Considering the values of c-statistic after bootstrap validation, the discriminative power of all prediction models was satisfactory, ranging from 0.686 to 0.776, indicating a medium to good ability to distinguish between cases and non-cases (Table 5). Calibration curves, corresponding Hosmer–Lemeshow tests, and E/O ratios were presented in S1 Appendix.

**Table 5:** C-statistics of prediction models after apparent and bootstrap validation.

C-statistic	Prediction models including systolic and diastolic blood pressure		
	M1 (n = 520)	M2 (n = 435)	M3 (n = 326)
Apparent	0.778 (0.735, 0.821)	0.694 (0.642, 0.746)	0.783 (0.728, 0.838)
After bootstrapping	0.776 (0.734, 0.829)	0.686 (0.634, 0.741)	0.744 (0.691, 0.795)
	**Prediction models including mean blood pressure**		
	**M1 (n = 520)**	**M2 (n = 435)**	**M3 (n = 326)**
Apparent	0.775 (0.732, 0.818)	0.695 (0.643, 0.746)	0.769 (0.711, 0.826)
After bootstrapping	0.773 (0.732, 0.826)	0.687 (0.636, 0.744)	0.735 (0.677, 0.785)

Data are expressed as c-statistic (95% confidence interval)

### Model presentation

We suggest M1 and M2, including MBP for predicting 5- and 10-year DR risk, respectively. Moreover, M3, including MBP is recommended to predict the five-year risk. Choosing between M1 and M3 can be based on the availability of predictors in clinical practice. Given that the models’ c-statistics after bootstrap validation were roughly similar, this recommendation was based on the models’ completeness and the c-statistic, which is a crucial metric for comparing predictive models. The algorithm of recommended models has the form of:

1. M1 including MBP to predict five-year risk:

ln $\frac{p}{1-p}$ = −5.2855+(0.1412×diabetes duration)+(0.0175×MBP)+(0.0070×BG)

1. M2 with MBP to predict 10-year risk:

ln $\frac{p}{1-p}$ = −4.2554+(0.0876×diabetes duration)+(0.0192×MBP)+(0.0048×BG)

1. M3 including MBP to predict five-year risk:

ln$\;\frac{p}{1-p}$ = 8.4990+(0.1827×diabetes duration)+(−0.0389×MBP)+(−0.8821×HbA1c)+(−0.3027×HDL-C)+(−0.0214×TG)+(0.0117×FBG)+(−0.0008×(diabetes duration×FBS))+(0.0345×(HbA1c×HDL-C))+(0.0002×(TG×MBP))

Where p is the risk of DR, and the right side of the algorithm is the linear predictor. Predicted absolute risk ($\hat{p}$) for a specific individual can be estimated by inserting his/her predictor values into the algorithm, then transforming back to the probability scale using the equation of $\hat{p}=\frac{exp\;(linear\;predictor)}{1+exp(linear\;predictor)}$. For example, an individual with T2DM for 10 years, at a routine clinical check-up has a MBP of 100.33 mmHg, and a BG of 301 mg/dL. Using M1, the probability that she/he will develop DR in the next five years is predicted to be 50%.

### Clinical usefulness

**Fig 2:**
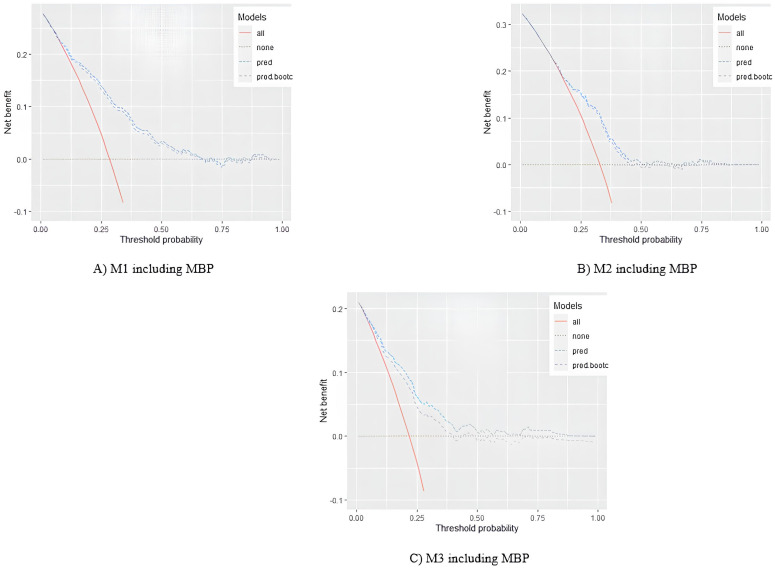
Decision curves of suggested prediction models. The y-axis indicates the net benefit. The x-axis indicates the thresholds of predicted probabilities. The solid line represented treat all strategy. The dotted line represented the treat none strategy. The dashed line (pred) describes the net benefits of the prediction model at various thresholds using apparent validation. The long-dashed (pred.bootc) line describes the bootstrap corrected net benefits of the prediction model at various thresholds.

Based on the decision curve of final suggested models after bootstrap validation, the interval of threshold probabilities in which M1 would be useful ranged from 0.12 to 0.60. The maximum value of Youden statistic occurred at a cut-off point of 0.24 which was equal to 0.45. Furthermore, the interval for M2 was 0.22 to 0.43. The maximum value of the Youden statistic occurred at a cut-off point of 0.32 which was equal to 0.43. Thus, the interval for M3 was 0.12 to 0.37. The corresponding maximum value of the Youden statistic occurred at the cut point of 0.20 which was equal to 0.45. The decision curves are shown in Fig 2.

## Discussion

This study aimed to develop a statistical model that can predict the risk of DR in 5 and 10 yearsˈ timeframe in the individuals with T2DM. Therefore, using data from a population-based prospective cohort study, we developed and validated six regression models including various predictors. Based on the availability of predictors in clinical settings, we discovered that three models could be recommended out of all the developed models: one for predicting 10-year risk and two for predicting 5-year risk. These three models showed that the duration of diabetes, BG, FBG, HbA1c, MBP, HDL-C, and TG levels were critical predictors of DR. Moreover, the models represented favorable predictive performance measures.

### Major predictors of DR

#### Disease course.

Previous studies established that a longer diabetes duration is a major non-modifiable risk factor for the onset of DR; a recent systematic review revealed that diabetes duration was associated with DR development with a pooled odds ratio (OR) of 1.03 (95% CI: 1.02, 1.03) [26]. Other previous epidemiological studies confirmed a relationship between long duration of diabetes and DR [27–29]. The present study discovered a statistically significant association between the length of diabetes and DR, which is consistent with previous research. The impact of chronic exposure to hyperglycemia, which causes microvascular damage, is probably the cause of this connection. However, because of the impossibility of intervention in this factor, it can only be considered a significant predictor to identify high-risk patients and instead focus on modifiable risk factors.

#### Blood glucose.

After the diabetes duration, hyperglycemia is reported to be the most relevant risk factor for the development of DR [30]. In a systematic review, and meta-analysis, 2-hour postprandial blood glucose, FBG, and HbA1c were reported to be associated risk factors of DR with the pooled OR of 1.94 (95% CI: 0.81, 4.65), 1.33 (95% CI: 1.12, 1.59), and 1.15 (95% CI: 1.09, 1.20), respectively [31]. The results of our study revealed that BG was a statistically significant predictor across all multivariable models where this variable was included. A similar pattern was observed in the case of FBG. These results are in agreement with those of prior research, suggesting that elevated blood glucose levels may contribute to the development of DR. Furthermore, HbA1c is a widely recognized glycemic control indicator, and its influence on the development of DR has been thoroughly examined in prior research [26,31,32]. Nevertheless, our investigation demonstrated a detrimental association. One possible explanation is that HDL-C has a protective effect, and the regression models included an interaction effect between HbA1c and HDL-C. Therefore, HbA1c may have different effects on DR depending on HDL-C levels.

#### Blood pressure.

Several studies suggested that high blood pressure can contribute to the development of DR. A large population-based cross-sectional study discovered an association between hypertension and DR with an adjusted OR of 1.08 (95% CI: 1.04, 1.11) [33]. Moreover, in another population-based study, poorly controlled and untreated hypertension were associated with DR with adjusted OR of 1.97 (95% CI: 1.39, 2.83), and 2.01 (95% CI: 1.34, 3.05), respectively. Systolic blood pressure was associated with DR with an adjusted OR of 1.45 (95% CI: 1.28, 1.65) [34]. The results of present study showed that SBP in M1, DBP in M2, and both variables in M3 were significantly associated with DR. These findings indicate the predictive capability of both variables. Therefore, prediction models were developed by combining these two variables as the MBP. Mean blood pressure was a statistically significant predictor of both M1 and M2.

#### Lipid profile.

Dyslipidemia is a common comorbidity of diabetes [35,36], and can be involved in the development of DR [37]. Research findings on the association between HDL-C and DR are conflicting. While some studies suggest that HDL-C plays a protective role against DR development [38], others indicate a positive association between HDL-C levels and DR occurrence [39]. Therefore, a study revealed an inverted U-shaped association between HDL-C and DR [40]. Our findings indicate a statistically significant protective effect of HDL-C on DR in univariable and multivariable regression models. There is a discrepancy among the studies regarding the association of TG and DR [37,41,42]. Triglycerides was identified as a detrimental risk factor in our investigation. A positive association between TC and DR has been substantiated by numerous investigations [37,43]. In contrast, the current study did not demonstrate a statistically significant relationship between TC levels in either univariable or multivariable analyses. This discrepancy may be due to the small sample size and consequent lack of statistical power to detect a significant association.

### Prediction models

#### Model development.

After incorporating the significant predictors from the univariable regression analyses of this study, three predictor variables, including diabetes duration, MBP, and BG constructed final suggested M1 and M2. In addition to these variables, HbA1c, FBG, HDL-C, and TG levels were involved in the development of suggested M3.

Several DR risk prediction models were developed and validated in various populations. A recent population-based cohort study in China developed a model to predict the risk of vision-threatening DR including predictors of age, BMI, SBP, diabetes duration, and HbA1c, with a c-statistic of 0.72 and 0.68, respectively, for the training and testing set, and a good calibration [44]. In addition, a model was fitted using diabetes duration, HbA1c, FBG, SBP, proteinuria, BMI, and education level, as determined by cross-sectional study data from Iran. The model exhibited a c-statistic of 0.76 and a high degree of agreement between the predicted and observed risk [7]. Another risk prediction algorithm by Aspelund et al. was created to calculate the risk of sight-threatening DR in a time period ranging from 6 to 60 months based on clinical data, including type and duration of diabetes, HbA1c, SBP, presence and DR grade, from an Icelandic type 1 and 2 population with diabetes. The c-statistic of the algorithm was found to be 0.76 [45]. A validation study by Rao et al., applying the data of T2DM patients from Australia, New Zealand, and Finland, showed that the Aspelundˈs algorithm predicted two- and five-year risk of sight-threatening DR well with a c-statistic of 0.86 and 0.86, respectively [46].

Several predictors in our risk prediction models are common to existing models, and their significance in DR development has been previously documented in the literature, as mentioned earlier.

#### Model performance.

Discrimination analysis of all models in this study, using the c-statistic, demonstrated acceptable power to discriminate between low- and high-risk individuals, even after correcting for optimism. The c-statistic shows the probability that, of two people, one will have DR within the next 5 or 10 years and the other will not, with the patient who will acquire DR having a larger predicted risk than the patient who will not. The discriminatory ability of prediction models in our study was consistent with that in previous evidence [7,44,45]. Including key predictors may be a reason for achieving moderate-to-good discrimination power in this study, and consistency with other studies. However, we observed lower discrimination than that in the model validated by Rao et al. [46], which may be due to differences among populations and outcome spectrum. Furthermore, in all the suggested models, the mean predicted values were close to the mean observed outcomes (E/O ratio close to 1), indicating good calibration, except in higher risks (graphically tested). The latter implies a relatively poor calibration at higher risks, which may be due to the lower number of observed DR cases.

#### Clinical usefulness.

In addition to discrimination and calibration, we assessed the ability of models to make better clinical decisions than settings without using the model. For M1, no difference was observed between the “treat all” and “treat none” strategies and the model line at predicted risk levels below 0.12 and above 0.6, with a proposed cut-off point of 0.24. Using this model in clinical settings, individuals with a predicted risk over 0.24 should be categorized as positive for developing DR in the next five years and need care, while other patients will be classed as negative. This cut-off point was thought of as a decision threshold. Moreover, due to M2, the appropriate threshold interval was found to be 0.22 to 0.43 with a proper cut-off point of 0.32 to classify patients based on 10-year predicted risk. Decision curve of M3 also showed that in the predicted risk of less than 0.12, there is no difference between “treat all” approach and the prediction model. Hence, in the predicted risk higher than 0.37, there is no difference between “treat none” and the model line. The most appropriate decision threshold for M3 was 0.20. Previously, a clinically useful predicted risk threshold interval of 21–51% by Mo et al. [47], 2–85% by Wang et al. [48], and 11–95% by Liu et al. [49] were proposed for Chinese population. However, to our knowledge, such an interval has not been reported in the Iranian population. We suggested the most suitable cut-off point based on statistical considerations to categorize people as future cases or non-cases of DR, but healthcare providers are free to select any other reasonable classification cutoff within the clinically useful interval based on the patient’s condition or the importance of false positives or false negatives.

### Strengths and limitations of the study

The strengths of this study include the development of predictive models using data from a prospective cohort study with 10-year follow up and a representative sample from the general population. Thus, easily obtainable predictors were considered to develop the models, which could facilitate their use in clinical settings. Furthermore, as far as we are aware, this is the first research to evaluate the clinical usefulness of a DR risk prediction model in the Iranian community in order to establish a cutoff point for categorizing people as positive or negative.

The study limitation was the diagnosis of diabetes based on BG. Another limitation was the unavailability of FBG, HbA1c, and lipid profile in the first phase of ShECS. Moreover, Determining the duration of diabetes based on the time since diagnosis may have been underestimated, because diabetes may have remained undiagnosed for some time. However, considering that most individuals are checked up periodically and the prominent symptoms of diabetes that lead people to refer, it is expected that the effect of this underestimation on the study results is small and negligible.

### Potential impact on clinical practice

Early identification of DR is crucial for effective treatment and preservation of vision [50]. Clinicians may use our prediction algorithms to identify high-risk individuals who might need extra care or intervention by using easily accessible clinical indicators. Consequently, preventive strategies can be enhanced by focusing the modifiable risk factors. Furthermore, determining the interval of risk thresholds allows healthcare providers and clinicians to select the optimal cutoff point for classifying individuals as potential future positive or negative cases within a narrowed, reasonable span of predicted probabilities.

### Conclusions

Three prediction models for DR risk were introduced using logistic regression analysis: two for 5-year risk and one for 10-year risk. Among five-year risk prediction models, one incorporated diabetes duration, MBP, and BG, while another included these factors, along with FBG, HbA1c, HDL-C, and TG measurements. Moreover, 10-year risk prediction model included diabetes duration, MBP, and BG. The models demonstrated sufficient predictive performance in terms of discrimination and calibration after internal validation. Through decision curves, the clinically useful interval of the predicted risk threshold was identified based on NB for each final model and a single cutoff point was proposed from a statistical perspective.

## Supporting information

S1 Appendix Calibration of the prediction models. The calibration plots indicate the mean of the predicted risks versus observed outcome proportions in deciles of the predicted risks. Both apparent and optimism-corrected calibration were reported. The 45° diagonal line is the line of identity that represents perfect predictions. Circles show predicted risk deciles. The bold line indicate the Lowess smoother line of agreement between observed and expected (predicted) risks.
(PDF)
