# Supplementary material for: Development and validation of prediction models for diabetic retinopathy in type 2 diabetes patients
Source: PLoS One. 2025 Jul 10;20(7):e0325814. doi: 10.1371/journal.pone.0325814 (PMC12244548; doi:10.1371/journal.pone.0325814)
Supplement: S1 Appendix — The calibration plots indicate the mean of the predicted risks versus observed outcome proportions in deciles of the predicted risks. Both apparent and optimism-corrected calibration were reported. The 45° diagonal line is the line of identity that represents perfect predictions. Circles show predicted risk deciles. The bold line indicate the Lowess smoother line of agreement between observed and expected (predicted) risks. (PDF) [file pone.0325814.s001.pdf]

S1 Appendix. Calibration of the prediction models. The calibration plots indicate the mean of the predicted risks versus observed outcome proportions in deciles of the predicted risks. Both apparent and optimism-corrected calibration were reported. The 45° diagonal line is the line of identity that represents perfect predictions. Circles show predicted risk deciles. The bold line indicate the Lowess smoother line of agreement between observed and expected (predicted) risks.

S1 Appendix-A. Prediction models including systolic and diastolic blood pressure

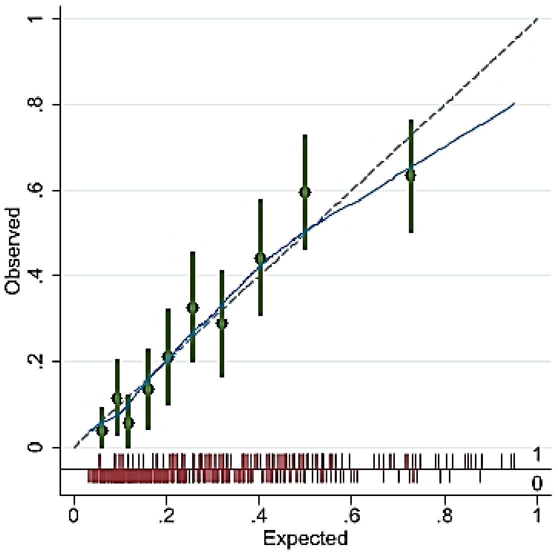

M1(a)

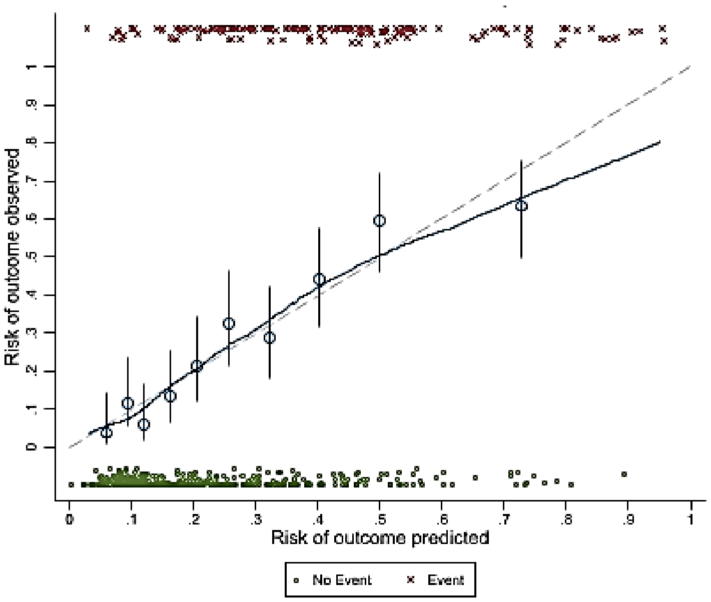

M1(b)

		Expected (predicted) to observed ratio (95% confidence interval)	Hosmer–Lemeshow statistic (p-value)
**Apparent**	**M1(a)**	1.00 (not available)	9.002 (0.34)
**Optimism-corrected**	**M1(b)**	0.997 (0.874, 1.137)	Not applicable

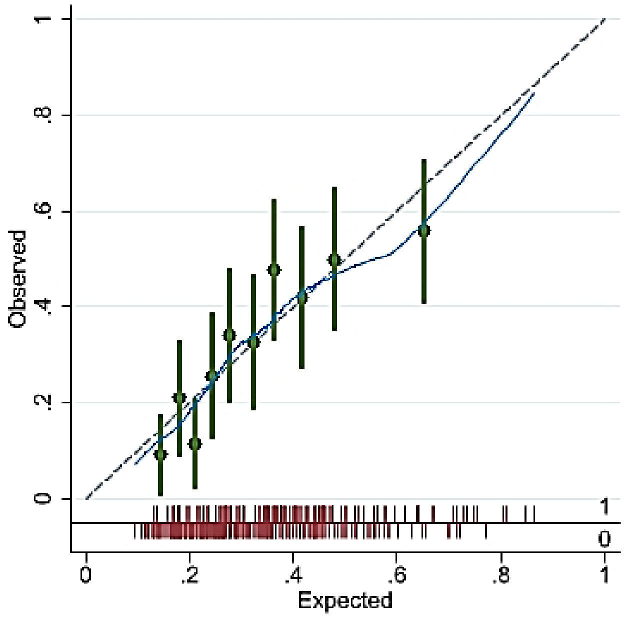

M2(a)

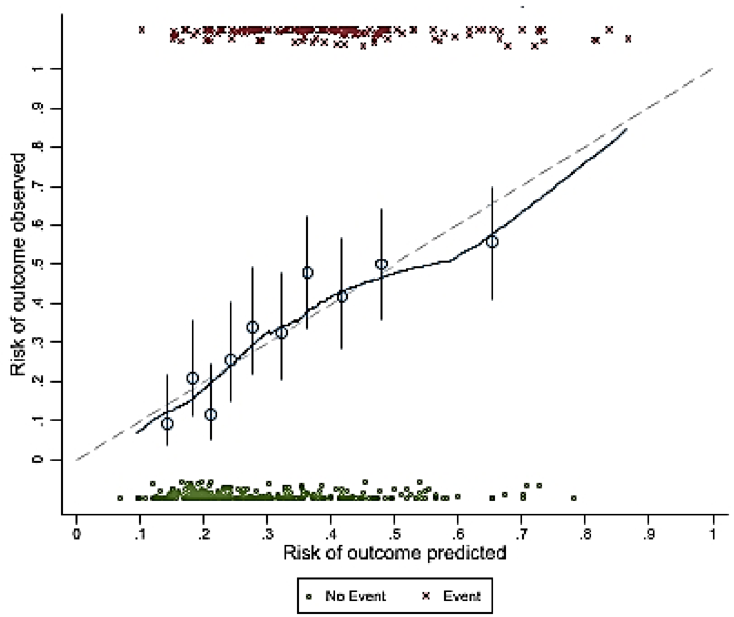

M2(b)

		Expected (predicted) to observed ratio (95% confidence interval)	Hosmer–Lemeshow statistic (p-value)
**Apparent**	**M2(a)**	1.00 (not available)	7.201 (0.51)
**Optimism-corrected**	**M2(b)**	0.996 (0.863, 1.140)	Not applicable

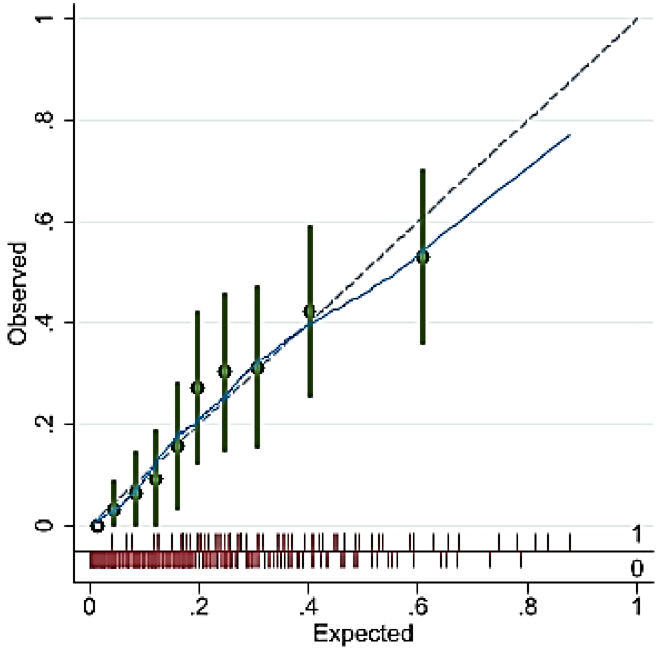

M3a

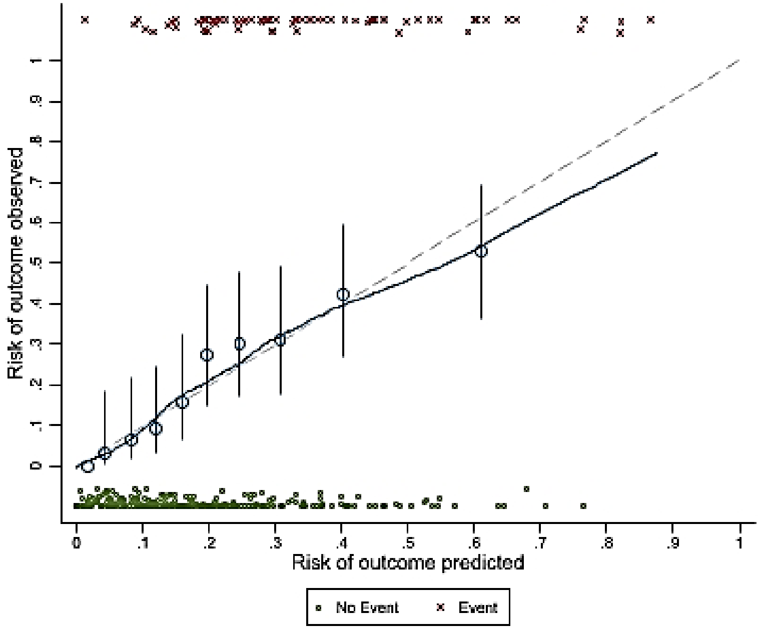

M3b

		Expected (predicted) to observed ratio (95% confidence interval)	Hosmer–Lemeshow statistic (p-value)
**Apparent**	**M3(a)**	1.00 (not available)	3.257 (0.92)
**Optimism-corrected**	**M3(b)**	1.007 (0.826, 1.211)	Not applicable

S1 Appendix-B. Prediction models including mean blood pressure

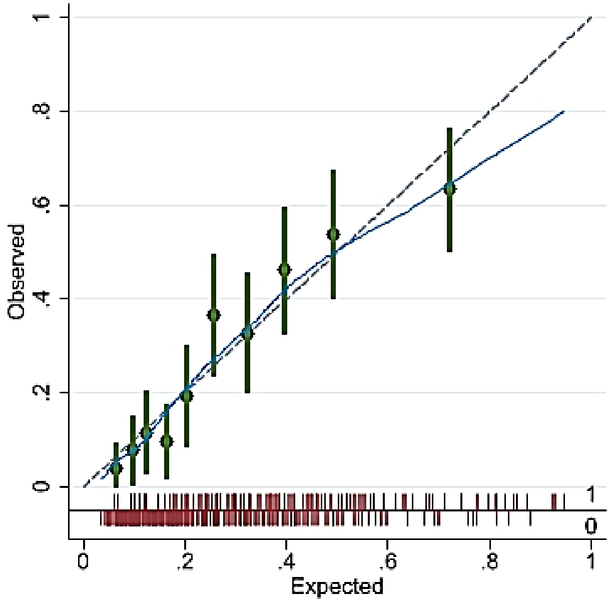

M1(a)

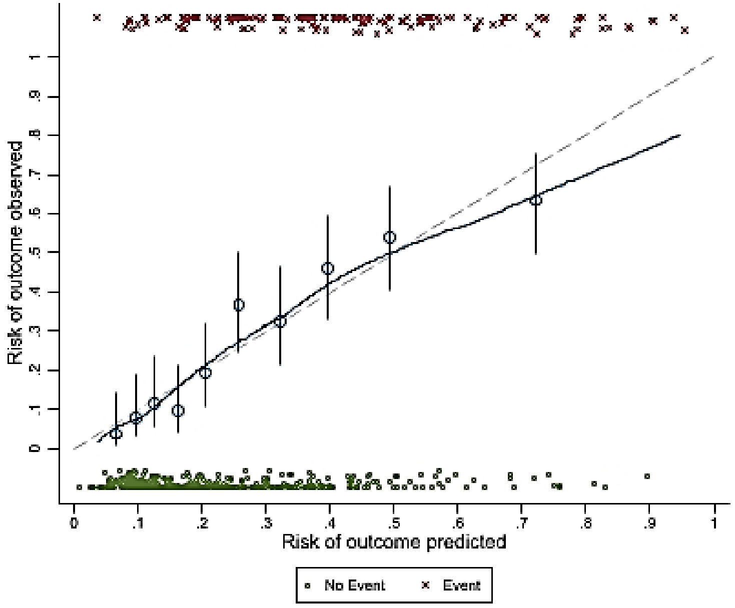

M1(b)

		Expected (predicted) to observed ratio (95% confidence interval)	Hosmer–Lemeshow statistic (p-value)
**Apparent**	**M1(a)**	1.00 (not available)	9.105 (0.33)
**Optimism-corrected**	**M1(b)**	0.997 (0.881, 1.133)	Not applicable

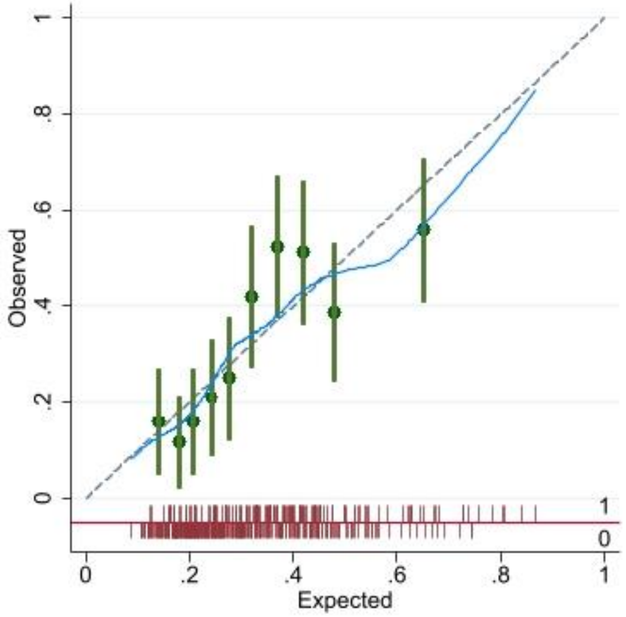

M2(a)

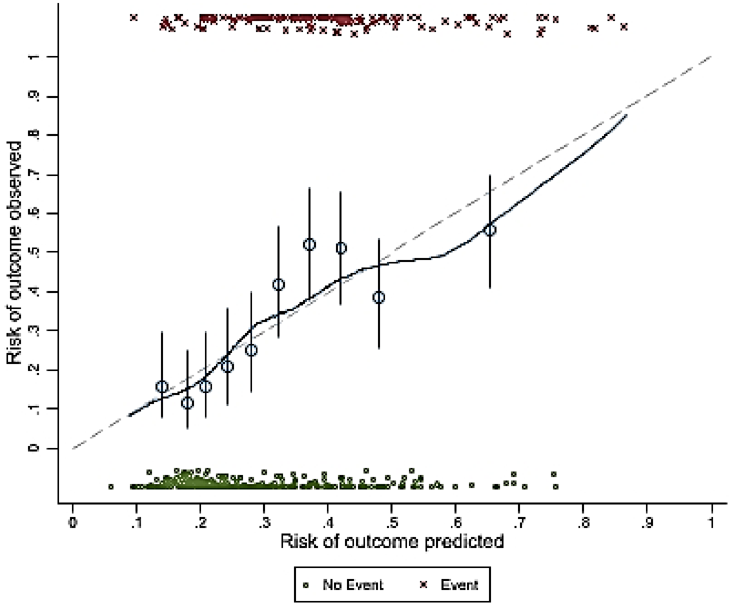

M2(b)

		Expected (predicted) to observed ratio (95% confidence interval)	Hosmer–Lemeshow statistic (p-value)
**Apparent**	**M2(a)**	1.00 (not available)	11.976 (0.15)
**Optimism-corrected**	**M2(b)**	0.996 (0.869, 1.143)	Not applicable

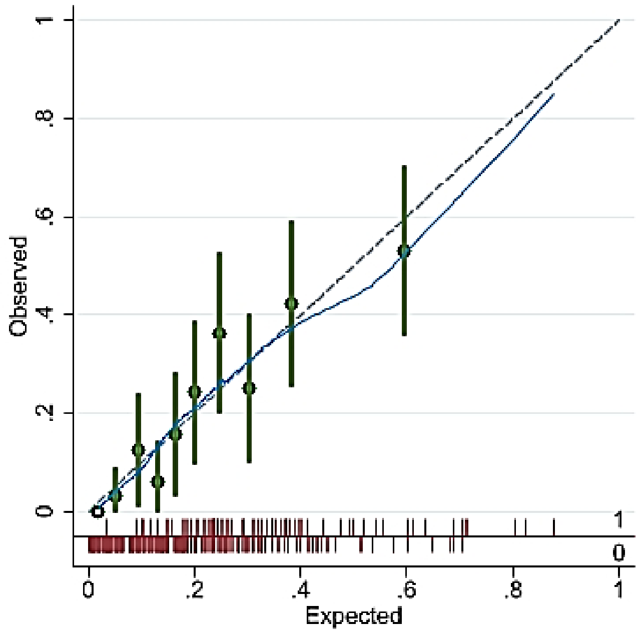

M3(a)

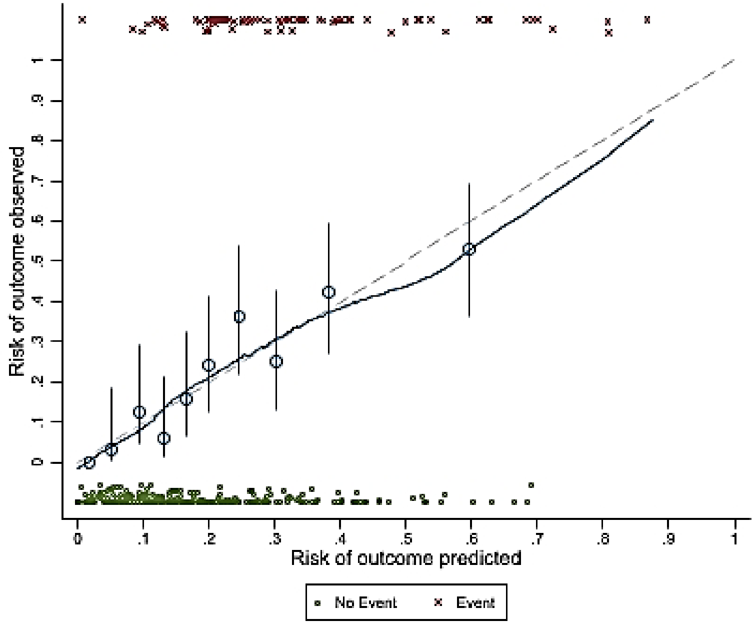

M3(b)

		Expected (predicted) to observed ratio (95% confidence interval)	Hosmer–Lemeshow statistic (p-value)
**Apparent**	**M3(a)**	1.00 (not available)	6.757 (0.65)
**Optimism-corrected**	**M3(b)**	1.005 (0.812, 1.201)	Not applicable
